# Caught Between External Pressures and Internal Battles: Psychosocial Factors Affecting Gestational Weight Gain – A Scoping Review

**DOI:** 10.7759/cureus.13487

**Published:** 2021-02-22

**Authors:** Unsa Athar, Noor Ul Ain Daud, Warda A Khan, Amna Khalid, Seemab Imtiaz Gill

**Affiliations:** 1 Community Health Sciences, Shalamar Medical and Dental College, Lahore, PAK; 2 Internal Medicine, King Edward Medical University, Lahore, PAK

**Keywords:** obesity，pre-pregnancy body mass index，gestational weight gain，cesarean section，macrosomia，perinatal outcomes, psychosocial factors, low socioeconomic, rate of gestational weight gain, weight gain in pregnancy, inadequate weight gain in pregnancy

## Abstract

Many physical factors (including maternal comorbidities) affecting gestational weight gain (GWG) have been widely studied; however, the psychosocial aspects pertaining to this need to be put under the microscope, especially in countries characterized by low indicators of socioeconomic development. Gaining and maintaining an adequate amount of weight during pregnancy is important to prevent premature deliveries, fetal demise, fetal macrosomia, shoulder dystocia during delivery, emergency cesarean sections, postpartum weight retention, childhood obesity, etc. A scoping review of the articles published in the last five years has revealed that perinatal outcomes like gestational weight are influenced by certain psychosocial factors, including, but not limited to, intimate partner violence, lack of social support and recognition, financial distress, household food insecurity, chronic stress and depression related to pregnancy, eating pathologies, and low self-esteem. Employing a multi-disciplinary approach, which involves seeking the help of psychiatrists/psychologists, obstetricians, nutritionists, and public health specialists, can help us mitigate undesirable outcomes related to inadequate and excessive weight gain during pregnancy. More intervention-based research focusing on psychosocial factors relating to GWG is needed in regions like South Asia, which is associated with low indicators of socioeconomic development.

## Introduction and background

Many factors contribute toward helping women maintain a healthy physical and mental state during pregnancy and successfully carry it to term, preferably without any complications for the mother and fetus alike. Factors affecting the physical health of the mother, and hence the fetus that is carried, such as the presence of underlying comorbidities like obesity, diabetes, hypertension, dyslipidemias, metabolic syndrome, or other physical diseases like systemic inflammation, vascular dysfunction, which might affect the mother in the pre-gestational and gestational periods, have been extensively studied [1]. However, the psychosocial factors responsible for maternal health and their potential impact on the well-being of both the mother and fetus remain largely understudied; hence, a clear association between them has not been established [2].

Regarding the evolution of medical perceptions toward gestational weight gain (GWG), clinicians used to believe that a weight gain of more than 9 kilograms can lead to detrimental effects [3]. In the early 1930s, more research was conducted on the subject, and by the 1970s, it was established that an adequate weight gain maintained by a healthy diet and activity is essential for favorable outcomes for the mother and the fetus [3]. The Institute of Medicine (IOM) and National Research Council (NRC) recommend an ideal GWG of 11.5-16 kilograms for women who have a normal pre-pregnancy weight/body mass index [4]. Gaining and maintaining an adequate amount of weight in pregnancy is important because the lack of it leads to premature deliveries owing to small-for-gestational-age weight in the babies, and even fetal demise. On the other hand, excessive weight gain also has numerous maternal and fetal repercussions, such as fetal macrosomia, shoulder dystocia during delivery, emergency cesarean sections, postpartum weight retention, and childhood obesity [5].

Even though many physical factors (including maternal comorbidities) affecting GWG have been studied, the psychosocial aspects of it need to be delved into in a deeper manner, especially in countries with low socioeconomic indices. A study performed in Pakistan [6] showed that depression experienced by mothers during pregnancy is chronic in nature and known to persist for as many as three to four years following childbirth. This period is considered crucial for the emotional and cognitive development of an infant, which can be adversely affected by the lack of mental well-being in mothers. Maternal depression can also lead to poor physical outcomes in children, such as stunting and underweight, emphasizing the influence of maternal mental health on the physical and psychological aspects of a child’s health [6].

With this context in mind, our objective in conducting this review was to scope through the existing evidence about psychosocial factors affecting maternal health, in order to emphasize the need for and understand the extent of further research that needs to be performed on this topic.

## Review

Methodology

**Figure 1:**
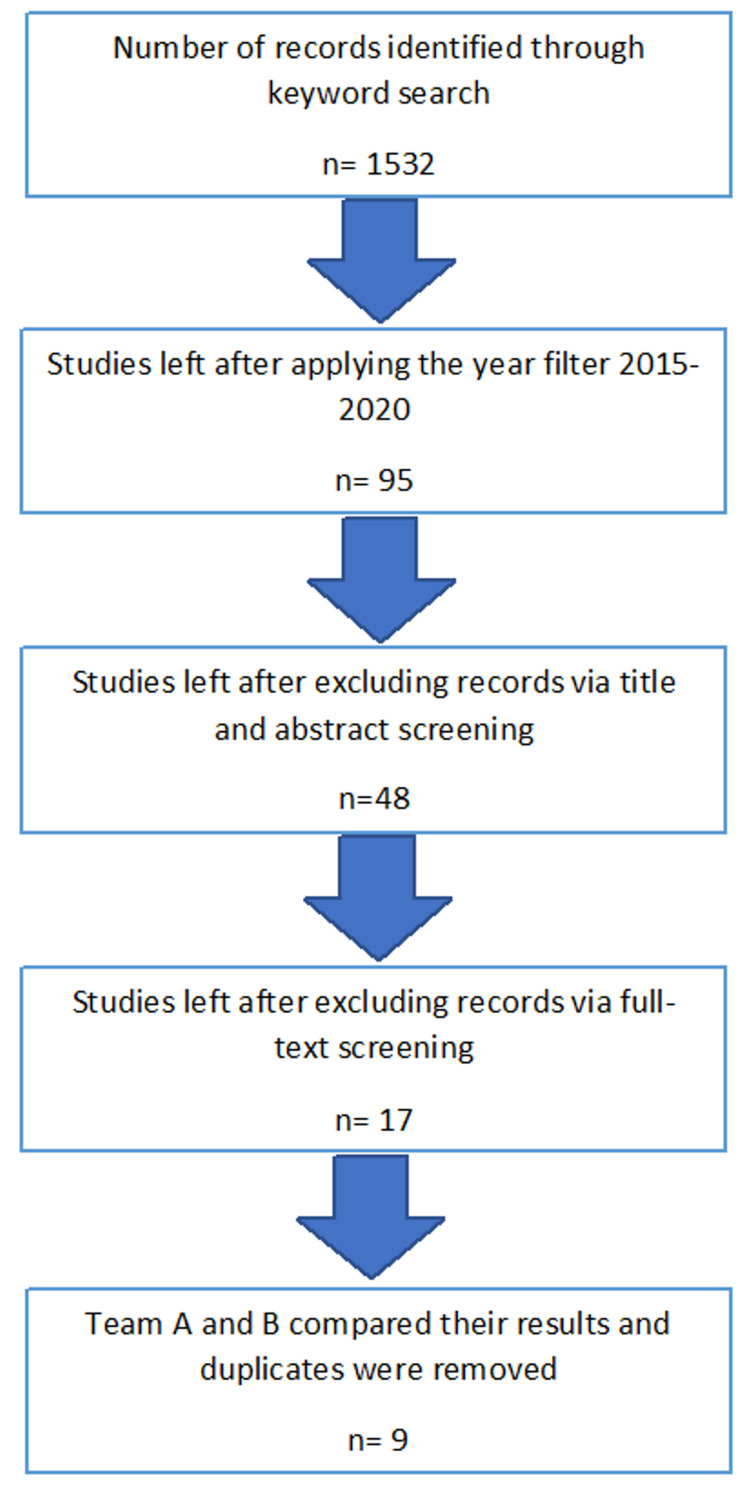
Selection of articles

**Figure 2:**
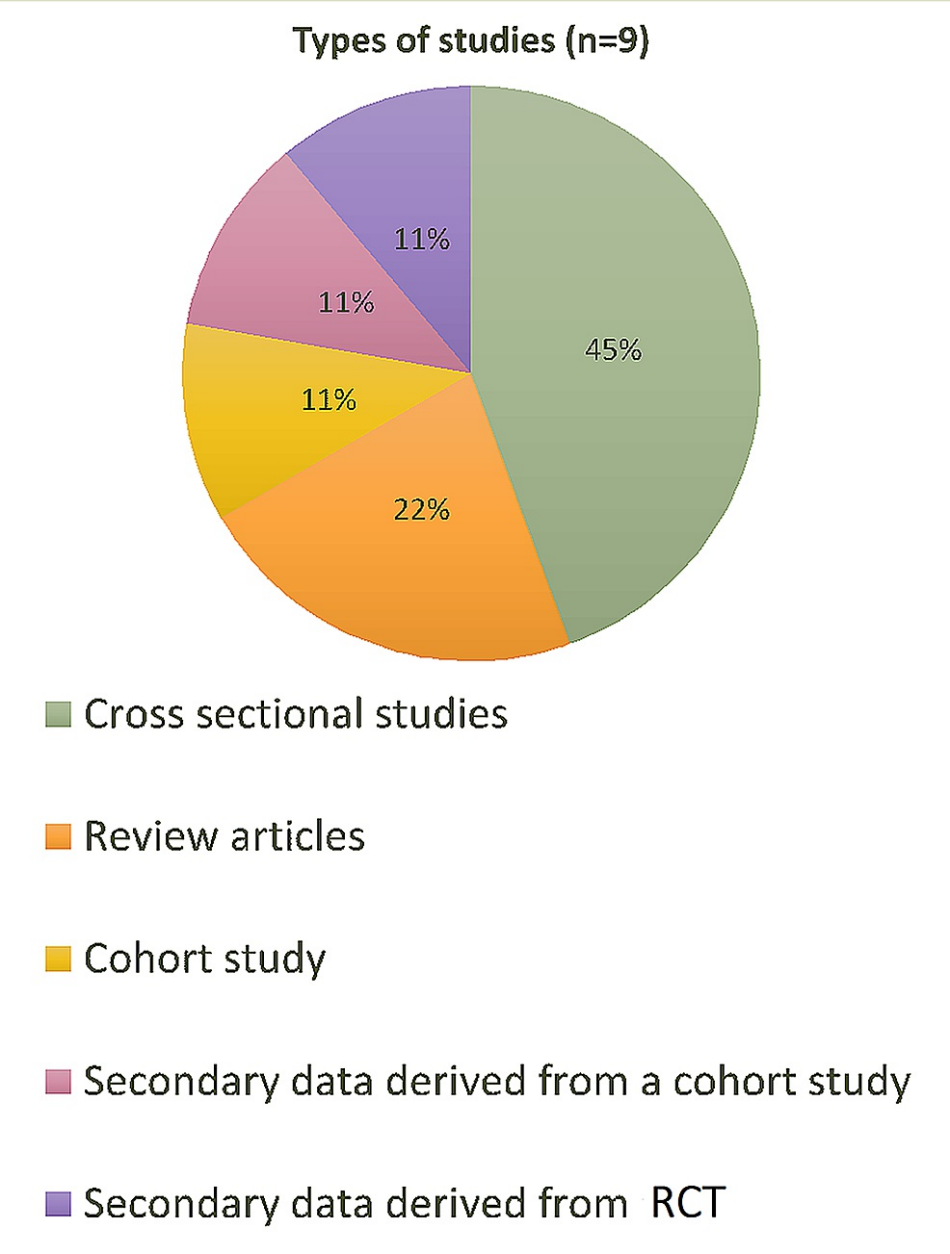
Type/methodology of selected articles (n=9) RCT: randomized controlled trial

An evidence-based methodological protocol [7] was employed to look for peer-reviewed articles. The authors were divided into two teams: A and B. Team A and Team B performed the first stages of the scoping separately. The following databases were used depending upon the ease of access: PubMed, PubMed Central, ProQuest, PsychINFO, Online Wiley Library, and Ovid. The Medical Subject Headings (MeSH) terms employed to conduct the search were as follows: ‘psychosocial factors AND gestational weight gain,’ ‘inadequate gestational weight gain,’ ‘excessive gestational weight,’ and ‘rate of weight gain in pregnancy.’ A total of 1,532 results were generated. A filter was then applied to select only those articles that were published from 2015 to 2020. The studies involving animals and those published in any language other than English along with repeated articles were eliminated. Articles that dealt with physical comorbidities concerning GWG, such as diabetes mellitus, as well as those involving women with a previous history of mental or psychiatric diseases, were excluded after reading through their methodologies (full-text screening). Studies involving previously diagnosed psychiatric or physical comorbidities were excluded so that their confounding effects on GWG could be removed. The population in the included studies had a singleton pregnancy, without any fetal anomalies. The two teams then matched and compared their results. Duplicates were removed and disagreements were resolved through discussions and by arriving at mutual agreements. All these factors narrowed down the number of concerned articles to a total of nine, the quality of which were assessed by using the Strengthening the Reporting of Observational Studies in Epidemiology (STROBE) [8] guidelines. This methodology adopted is illustrated in Figure 1. The articles that were ultimately included for analysis (n=9) were thoroughly studied by the authors. The focus was placed more on the methodology than the results as this was a scoping review. The different types of studies that were included are shown in Figure 2.

Results and discussion

**Figure 3:**
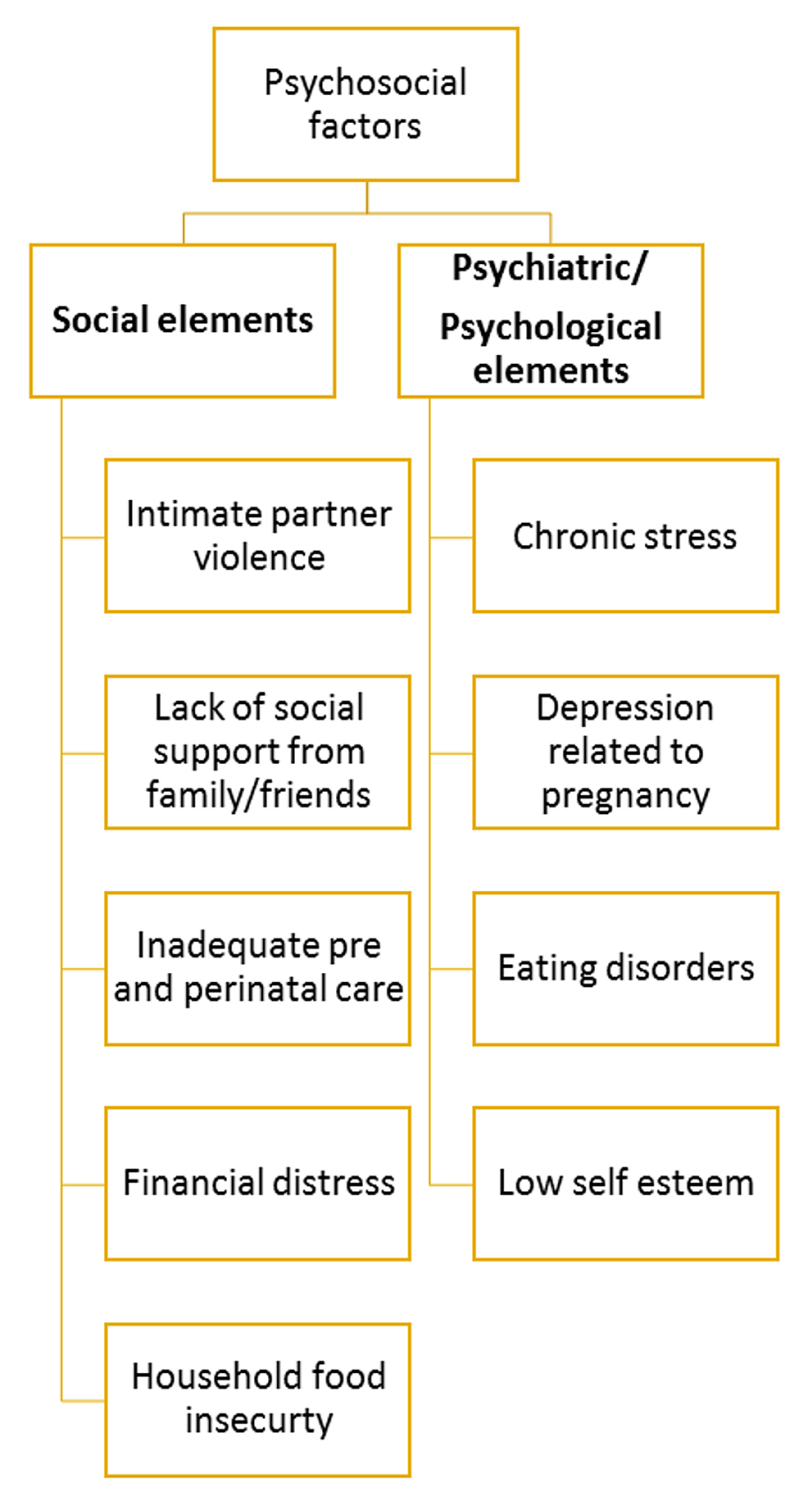
Psychosocial factors related to the inadequacy and/or excess of gestational weight gain

The primary outcomes (as depicted in the titles) that were studied, the bio-statistical tests applied, the population included in the articles [9-15], and their limitations are summarized inTable 1. There was a consensus among all the articles that there is a dearth of research on the impact of psychosocial factors and their relation with gestational weight. The IOM and NRC in 2009 declared that there is a dire need for research that can determine the interventions aimed at psychosocial elements leading to excessive or inadequate weight gain [4]. Almost a decade later, we are still facing a situation where there is a significant lack of work done on the aspect of psychosociology, as there still remains a stigma concerning mental health associated with cultural norms. To get an idea of where we stand today, this scoping review focused on the research methodology practiced in the last five years, in order to lay the groundwork for further intervention-based studies. The picture that has emerged after analyzing the selected articles clearly exhibited women caught between a society that is unwilling to provide support and the distortions of their own cognition by which they view themselves. Psychosocial factors that determine the inadequacy and/or excess of GWG can be classified into various types, as shown in Figure 3.

**Table 1:** Overview of the articles reviewed PRISMA: Preferred Reporting Items for Systematic Reviews and Meta-Analyses

Article	Population included	Primary outcomes studied	Primary bio-statistical tests applied to the data	Limitations
Hecht et al. [9]	N=70. Age: ≥18 years. At any stage of pregnancy	Eating pathology and depressive symptoms as predictors of excessive weight gain during pregnancy	Correlations, logistical regressions, multivariate regressions	Primarily Caucasian, married/partnered women with a graduate degree
Braig et al. [10]	N=748. Age: majorly between 26-35 years. Throughout pregnancy	Psychosocial stress and longitudinally measured gestational weight gain throughout pregnancy	Regression analysis	High socioeconomic status; self-reported stress and related constructs at the point just after delivery
de Jersey et al. [11]	N=664. Age: 29 ±5. At 16 ±2 weeks of gestation	Factors related to lifestyle health behaviors and weight gain in healthy and overweight pregnant women	Independent t-test, Mann-Whitney U test, Pearson/Spearman’s coefficient calculation, Pearson’s chi-square test	Recall bias, test-retest reliability not assessed
Hartley et al. [12]	Review article. N=12. Age not given. 15 weeks to >36 weeks of gestation	Psychosocial risk factors for excessive gestational weight gain	Qualitative analysis using PRISMA guidelines	Small number of psychosocial factors studied
Hartley et al. [13]	N=256. Age: >18 years. Gestation: <16 weeks	The effect of parity on psychosocial factors related to excessive gestational weight gain	t-test	Self-constructed (not validated) questionnaire
Dolin et al. [14]	N=50. Age: ≥18 years. Self-identified as Hispanic. At 28-32 weeks of gestation	Sociodemographic characteristics, health behaviors, and psychosocial stressors in pregnant women	Multinomial logistic regression, linear regression	Social desirability bias, recall bias
Mathews et al. [15]	N=1,073. Age: 28.7 ±4.6 years. ≥8 weeks' gestation	The role of mindfulness in psychosocial predictors of weight gain	Bivariate correlations, multiple regression analysis	Does not prove causation, recall bias as self-reported constructs were studied
Dolatian et al. [16]	N=73. Age: ≥18 years. 24-28 weeks of gestation	Predictors of weight gain in pregnancy	Chi-square/Fisher's coefficient calculation	A large number of questionnaires, which took a lot of time for patients to fill in
Plante et al. [17]	Narrative review. N=77. Age and gestational week not given	Relationship between psychosocial factors, dietary intake, and gestational weight gain	Qualitative analysis using PRISMA guidelines	The factor of physical exercise was not studied. The quality of articles was not systematically assessed

Social elements

Intimate Partner Violence and Lack of Social Support From Loved Ones

An analysis of variables related to pre-pregnancy BMI revealed that women who reported a lack of social support showed signs of improper GWG [13,14]. During their pregnancy, women felt isolated from friends and family, describing themselves as ‘not being appreciated adequately in social circles.’ They also experienced a stressful environment created by their neighbors' constant interference in their lives [14]. In addition to that, violence committed by intimate partners also bred an incompatible environment for a healthy pregnancy, which consequently impacted the rate of weight gain in pregnancy [15]. Dolatian et al. [16] created a conceptual framework showing the role of violence, giving it a central role in the rate of weight gain in pregnancy. Violence at the hands of intimate partners and other family members, particularly during pregnancy, has been detrimental to women's well-being since the advent of time [17]. Violence can be in different forms, including physical, sexual, and emotional. A study in Malaysia has reported that women who encounter violence during pregnancy show a higher odds ratio for developing anemia, inadequate weight gain, urinary tract infections, premature rupture of membranes, and antepartum hemorrhage [18].

Financial Distress and Household Food Insecurity

Through various analytical methods, it was observed that financial distress leads to statistically significant effects (p<0.05) on the rate of GWG [10,14]. Inadequate consumption of healthy food due to poor socioeconomic status (food insecurity) and the added burden of stress related to unemployment (inability to pay rent/bills) acted as a barrier for a healthy GWG [14]. Such perceived distress due to financial burden was also found to be related to both excessive (p=0.02) and inadequate weight gain in a study performed as a secondary data analysis of the Gestational Diabetes’s Effects on Moms trial conducted at Kaiser Permanente - Northern California (KPNC).

Psychiatric/psychological elements

Despite having no previously diagnosed psychiatric illness, many women experience depression, anxiety, and stress during pregnancy, which impacts their weight gain [9-13,15,16].

Chronic Stress and Depression

Stress has been long known as one of the many culprits behind excessive weight gain. Stress, particularly during pregnancy, disrupts the functioning of the hypothalamus-pituitary axis (HPA). This in turn increases the level of cortisol in the body, which redistributes fatty acid deposition to the center, and also increases ‘cravings’ that lead to over-eating [19,20]. Depressive symptoms and chronic stress have been unanimously found to be a predictor of excessive and/or inadequate weight gain [9-16]. Dolin et al. have reported that 11% of pregnant Hispanic immigrants showed perinatal depression and excessive weight gain, as assessed by a validated Patient-Health Questionnaire-9 tool [14]. The relationship between depression and GWG is, however, complicated and needs to be analyzed and studied more so that timely interventions and screening measures can be implemented. It is noteworthy that the practice of mindfulness techniques has shown an inverse relationship with the development of excessive weight gain [15]. The practice of mindfulness techniques by pregnant women has been observed to help overcome many hurdles like physical discomfort and psychological distress [21]. Getting a handle on mindfulness practices can help pregnant women become more self-aware of their condition, which in turn can help them keep their weight in check based on IOM guidelines.

Eating Disorders

Eating pathologies are also associated with excessive GWG worldwide [9]. Many pregnant women go through a phase of ‘emotional eating’ and cravings, mostly because of increased cortisol levels [10], which can lead to weight gain in excess of what is recommended by IOM. Dolin et al. [14] have reported a lower risk of excessive GWG in women who ate breakfast daily during their pregnancy. More than three hours of screen time and skipping breakfast turned out to be predictive of improper weight gain. Food insecurity is defined as the inability to access nutritious food due to poor living conditions and low economic status. In diverse urban populations, every one in 10 women is affected by food insecurity during their pregnancy [22]. The condition is even worse among rural populations and those living in urban slums. This food insecurity contributes to fostering eating pathologies [16]. Other factors that were found to cause eating disorders included low social support and unhappy marital relationships [17]. Following a healthy, balanced diet plan leads to a positive influence on the overall quality of life during pregnancy and thus ensures adequate weight gain [23]. To ensure that pregnant women get the full benefit of a healthy diet, psychosocial factors that contribute to eating disorders should be managed in a timely manner.

Low Self-Esteem

Women exhibiting sufficient self-efficacy were observed to experience adequate weight gain. Having a positive attitude towards weight management also showed an association with proper GWG [11]. Pregnant women who displayed low self-esteem and greater dissatisfaction with their bodies, characterizing themselves as ‘fat’ or ‘unattractive’, showed a higher risk of inadequate weight gain leading to undesirable perinatal outcomes [17]. The above observations clearly indicate the role psychosocial factors play in mediating an adequate GWG. Interventional approaches by obstetricians and physicians targeted to tailor to these psychosocial factors can improve maternal and neonatal outcomes. There is an apparent dearth of such research in South Asian countries, where psychosocial factors are much more prevalent compared to first-world countries. Social ills like child marriage have been shown to drastically affect adequate GWG. The prevalence of child marriage is high in Southeast Asia, the highest incidence being recorded in Bangladesh where approximately 52% of the girls are married before the age of 18 and 18% are married before 15 years of age. India is at a close second (47%), followed by Nepal (37%), Afghanistan (33%), and Pakistan (21%) [24]. A study on child marriages in Bangladesh has proposed that public health interventions aimed towards ending child marriages can bring about good perinatal outcomes, as child marriages were associated with inadequate gestational weight in the second and third trimesters [25].

Recommendations

Public health specialists need to be consulted in order to implement strategies at government levels to target the social elements discussed in this article. The availability of nutritious food and access to healthcare services need to be ensured. The spirit of social support must be fostered by providing the services of family counseling. Implementing strict laws by law enforcement agencies can be helpful in preventing child marriages and intimate partner violence. The plan of action for these recommendations is presented in Table 2.

**Table 2:** Recommendations and plan of action

Recommendation	Plan of action
Easy access to holistic healthcare services for pregnant patients	Training more birth-attendants and lady health workers/visitors in the fields of mental health and nutrition
Easy access to nutritious food	Availability of ration cards for pregnant females to buy fortified foods from utility stores
Availability of counseling	Free-of-cost appointments with psychiatrists and psychologists to help deal with stress/anxiety/depression/eating disorders related to or occurring in pregnancy in a timely manner
Public health interventions	More quantitative research (like cohort studies and randomized controlled trials) is needed after the implementation of the above
Availability of safe spaces	Functional shelters must be available for pregnant females experiencing abuse from an intimate partner

## Conclusions

A narrative review of the articles published in the last five years (2015-2020) has revealed the scope of research on GWG. Inadequate and excessive weight gain during pregnancy can have detrimental effects on both the mother and the child. Based on our findings, GWG is influenced by certain psychosocial factors, including, but not limited to, intimate partner violence, lack of social support and recognition, financial distress, household food insecurity, chronic stress and depression related to pregnancy, eating pathologies, and low self-esteem. Employing a multi-disciplinary approach, which involves using the services of psychiatrists/psychologists, obstetricians, trained birth attendants, nutritionists, and public health specialists, can help us mitigate the undesirable outcomes of inadequate and excessive weight gain. More intervention-based research targeting psychosocial factors is needed in regions characterized by low socioeconomic indicators, such as South Asia.
